# Supplementary material for: Associations between dietary patterns and intestinal inflammation among HIV-infected and uninfected adults: A cross-sectional study in Tanzania
Source: PLoS One. 2024 Dec 30;19(12):e0311693. doi: 10.1371/journal.pone.0311693 (PMC11684719; doi:10.1371/journal.pone.0311693)
Supplement: S1 Table — (DOCX) [file pone.0311693.s001.docx]

| Table: Food groupings used in dietary patterns analyses | | |
| --- | --- | --- |
| Group No. | Group Name | Macronutrients |
|  | Refined Grains | Cookie, Donut-African, Vermicelli, Biscuit, Donut, white bread roll, Macaroni or spaghetti, Pancake, uncooked, Cerelac, , Cake, fermented porridge |
|  | Unrefined Grains | Rice, whole grain stiff porridge (Maize, Sorghum ,millet) |
|  | Mixed Dish Grains | Rice biriyani, meat pillau, Mixed porridge flour (maize and oil), Rice and mung dish. |
|  | Natural Fruits and Juices | Guavas, Pineapple, Durian, ripe banana, ripe, Plums, Watermelon, Avocado, Custard-apple (bullock’s heart), Apple, Papaya, Peach, Date, Grapefruit, Jackfruit, Raspberry, Halwa, Baobab, Grapes, Orange, Tamarind, Tangerines, Lemon, Soursop, Mango, Pears, Passion, Limes, Cucumber, fruit juices |
|  | Artificial Sweetened Beverages | Carbonated beverage, Coca Cola, Blackcurrant syrup, Ribena, Mixed fruit jam, concentrated Fruit flavored drink, Ice sherbet, concentrated Orange juice |
|  | Legumes | Kidney bean, Chickpea (besan), Pigeon pea, Cow pea, Hyacinth beans, mung Bean, Lentil, French Bean |
|  | Red Meat | Beef, Mutton, meat, Goat meat |
|  | Chicken Meat | Chicken |
|  | Fish | Fresh water Fish, sea water fish, Small dried fish, sardines, high fat fish |
|  | Milk | Milk powder, full-cream, condensed Milk, Lactogen (infant formula), whole milk, plain Yogurt |
|  | Eggs | Boiled Egg boiled, Omelet, Spanish Egg, Fried Egg, Egg yolk, Omelet |
|  | Pork | Pork, medium fat, cooked |
|  | Banana Dishes | Banana with meat , boiled Banana, Banana porridge, plain, Banana with coconut milk, fried Banana, Banana with kidney beans, Banana with cashew nut, Banana with oil (no meat) |
|  | Potato Dishes | Boiled Sweet potato, boiled Potato, Potato samosa, Potato relish with coconut milk |
|  | Chips and Crisps | Potato chips, fried Sweet potato, Kachori (spicy potato balls), Cassava crisps or chips, Potato crisps |
|  | Yams | Taro, cooked, without salt, Taro, raw, Yam, raw |
|  | Pumpkins | Squash, Pumpkin |
|  | Cassava Dishes | Cassava with coconut milk, boiled Cassava, Cassava stiff porridge |
|  | Green Vegetables | Okra, Spinach, green Chili, Potato leaf relish, Cowpea leaf, Green leaf, Pumpkin leaf, Cassava leaf broth without oil , Green peas, Chinese Cabbage |
|  | Cruciferous vegetables | Cabbage, onion, Mushroom, Cauliflower |
|  | Dark_orange/Yellow vegetables | Carrots |
|  | Tomatoes | Tomato paste, Tomato ketchup, bitter Tomato bitter (African eggplant), Tomato ripe, green Tomato, Tomato relish |
|  | Alcohol | Commercial beer, local beer, wine |
|  | sweets and desserts | Candy, chocolate |
|  | Edible Insects | Termites, grasshopper |
|  | Honey and sugar | Honey, sugar, coffee/tea/milk with sugar |
|  | coffee | Coffee without sugar |
|  | Mixed dish tubers | Roots and tubers mixed Eg.Banana porridge with potatoes meat and spinach |
|  | Tea | Tea without sugar |
|  | seeds and nuts | Bambara nut, Sesame seed, immature Coconut, Almonds,  Coconut milk, Groundnuts, Pumpkin seed, Sunflower seed, Peanut butter, Cashewnut, Palm seed |
