# Supplementary material for: Associations between dietary patterns and intestinal inflammation among HIV-infected and uninfected adults: A cross-sectional study in Tanzania
Source: PLoS One. 2024 Dec 30;19(12):e0311693. doi: 10.1371/journal.pone.0311693 (PMC11684719; doi:10.1371/journal.pone.0311693)
Supplement: S2 Table — (DOCX) [file pone.0311693.s002.docx]

| **Table 2:** Factor loadings of the factors retained by principal component analysis and reduced rank regression analysis-derived dietary patterns | | | |
| --- | --- | --- | --- |
|  | **Vegetable-rich pattern** | **Vegetable-poor pattern** | **Carbohydrate-dense pattern** |
| Food group | Factor loadings | | |
| Unrefined grains | 0.0317 | 0.0529 | **0.7218** |
| Natural fruits and juices | **0.2889** | 0.0559 | 0.0890 |
| Artificial sweetened beverages | -0.0970 | **0.4073** | -0.0030 |
| Red meat | 0.1039 | **0.3176** | 0.0037 |
| Milk | 0.0685 | **0.2757** | 0.0006 |
| Banana dishes | **0.3004** | 0.0617 | 0.0010 |
| Potato dishes | **0.2914** | 0.0190 | -0.0320 |
| Chips and crisps | 0.0774 | **0.2954** | 0.0169 |
| Green vegetables | **0.3086** | 0.1111 | 0.0599 |
| Cruciferous vegetables | **0.2576** | 0.0837 | -0.0020 |
| Dark orange-yellow vegetables | **0.3359** | 0.1266 | 0.0004 |
| Tomatoes | **0.2681** | **-0.2540** | 0.0370 |
| Alcohol | 0.1389 | **0.3630** | 0.0329 |
| Foods with factor loading of ≥ \|0.25\| in any of the 3 patterns have been presented in this table. | | | |
