# Supplementary material for: Associations between dietary patterns and intestinal inflammation among HIV-infected and uninfected adults: A cross-sectional study in Tanzania
Source: PLoS One. 2024 Dec 30;19(12):e0311693. doi: 10.1371/journal.pone.0311693 (PMC11684719; doi:10.1371/journal.pone.0311693)
Supplement: S3 Table — (DOCX) [file pone.0311693.s003.docx]

| **S3 Table: Pair-wise correlation of the markers of inflammation** | | | | |
| --- | --- | --- | --- | --- |
|  | Fecal myeloperoxidase | Fecal neopterin | Plasma C-reactive protein | Plasma lipopolysaccharide binding protein |
| Fecal myeloperoxidase | 1.000 |  |  |  |
| Fecal neopterin | 0.008 | 1.000 |  |  |
| Plasma C-reactive protein | 0.017 | -0.030 | 1.000 |  |
| Plasma lipopolysaccharide binding protein | 0.044 | 0.025 | 0.311 | 1.000 |
