# Supplementary material for: Associations between dietary patterns and intestinal inflammation among HIV-infected and uninfected adults: A cross-sectional study in Tanzania
Source: PLoS One. 2024 Dec 30;19(12):e0311693. doi: 10.1371/journal.pone.0311693 (PMC11684719; doi:10.1371/journal.pone.0311693)
Supplement: Attachment — Submitted filename: Rebuttal letter.docx [file pone.0311693.s004.docx]

To,

PLOS ONE Editorial Office

**Subject: Rebuttal to Manuscript Rejection - PONE-D-24-25042**

Thank you for your observation regarding the ethical approvals for our manuscript, "Associations between dietary patterns and intestinal inflammation among HIV-infected and uninfected adults: a cross-sectional study in Tanzania" (PONE-D-24-25042). We acknowledge the importance of meeting ethical standards for human subjects’ research and would like to provide clarification on the ethical clearances obtained for our study.

1. The first ethical clearance, dated December 2018 (NIMR/HQ/R.8a/Vol IX/2973), was mentioned in the ethical statements of our manuscript. However, I regret that I did not upload this document during the initial submission.
2. The second ethical clearance is from the Joint Research Ethics and Review Committee of the Catholic University of Health and Allied Sciences and Bugando Medical Centre (CREC/542/2022). This clearance was a requirement of my university, as every student must obtain ethical clearance before full registration into a PhD program. My enrollment in the study occurred prior to my PhD enrollment, which is why the university clearance was dated after the data collection.

We have now uploaded both ethical clearance documents accordingly.

We hope that this clarification resolves the concerns raised regarding the ethical approval process for our study. We respectfully request that you reconsider our manuscript for publication in PLOS ONE. Our study provides valuable insights into the associations between dietary patterns and intestinal inflammation in HIV-infected and uninfected adults, which could contribute significantly to the existing body of knowledge and inform future research and public health strategies in similar contexts.

Thank you for your time and consideration. We look forward to your favorable response.

Sincerely,

Dr Malindisa
