# Supplementary material for: Associations between dietary patterns and intestinal inflammation among HIV-infected and uninfected adults: A cross-sectional study in Tanzania
Source: PLoS One. 2024 Dec 30;19(12):e0311693. doi: 10.1371/journal.pone.0311693 (PMC11684719; doi:10.1371/journal.pone.0311693)
Supplement: Attachment — Submitted filename: Rebuttal PLOS ONE.docx [file pone.0311693.s005.docx]

To;

Sepiso K. Masenga, PhD
Academic Editor
PLOS ONE.

We would like to express our sincere gratitude to you and the reviewers for their valuable feedback on our manuscript numbered **PONE-D-24-26062** titled “Associations between dietary patterns and intestinal inflammation among HIV-infected and uninfected adults: a cross-sectional study in Tanzania” submitted to the PLOS ONE for publication. We appreciate the time and effort invested in evaluating our work. Following the reviewers' constructive comments, we have revised the manuscript accordingly, and we believe that the revised version addresses the concerns raised during the review process.

We have addressed each of the reviewers' comments individually in the following point-by-point rebuttal:

.

| Comment | Response | Reference lines |
| --- | --- | --- |
| Editor   Please ensure that your manuscript meets PLOS ONE's style requirements, including those for file naming | Thank you, we have done that as advised |  |
| Please state what role the funders took in the study | Thank you, we have stated in the cover letter that, the funders had no role in study design, data collection and analysis, decision to publish, or preparation of the manuscript | Cover letter |
| Please expand the acronym “EDCTP” | Thank you, we have now expanded as advised | Cover letter, Manuscript lines 264 |
| Please amend your list of authors on the manuscript to ensure that each author is linked to an affiliation | Thank you,  This was confirmed |  |
| Please include captions for your Supporting Information files at the end of your manuscript, and update any in-text citations to match accordingly | Thank you,  This has been done accordingly | 253-256 |
| Please review your reference list to ensure that it is complete and correct | Thank you,  We have confirmed | References |
| Reviewer #1:  This manuscript explores a relevant topic of the link between diet and inflammatory markers. What is especially important is that it analyses diet as dietary patterns instead of individual nutrients and food groups which better reflects actual eating patterns and this is commendable. | We appreciate your dedication to review and contribution to the improvement of this manuscript |  |
| My biggest issue or point for clarification is the fact the authors make a distinction between HIV infected and uninfected individuals in the title, however this then not addressed in the manuscript. I would appreciate if the authors could clarify. | Thank you for highlighting this important point. We acknowledge the concern regarding the mention of HIV-infected and uninfected individuals in the title, while this distinction was not addressed in the manuscript. Initially, our study aimed to explore whether HIV status modifies the association between dietary patterns and inflammation, as inflammation is a key factor in both HIV progression and metabolic dysfunction.  Upon performing the analyses, we found no significant interaction between HIV status and the association between dietary patterns and inflammation. Consequently, we chose not to stratify the results by HIV status and instead adjusted for HIV as a confounding variable in the models  However, this cross-sectional study was nested in a cohort with HIV-positive and negative participants,  We thought having HIV in the title could best characterize our study participants.  We have added a text in results section to explain this. | 172-173 |
| Introduction: This section would benefit from a better explained rationale that is followed by the aims of the study. This can then be followed by a hypothesis but the aim should not be omitted. | We have revised the introduction to address this. | 77-85 |
| Also, the title clearly makes a distinction between HIV infected and uninfected individuals but this is not addressed in the introduction. There should also be a rationale provided for this. | We have responded this in the first comment, and the rationale have been provided. | 80-83 |
| Methods and results: I do not see any distinctions or analyses made to explore if there would be differences in results between HIV+ and HIV- participants, apart from Table 3. If this is indeed one of the objectives, then it should be addressed. This should then also be highlighted in discussion | We have responded this in the first response. | 172-173 |
| Reviewer #2  The present study titled “Associations between dietary patterns and intestinal inflammation among HIV-infected and uninfected adults: a cross-sectional study in Tanzania” has been conducted. Inflammatory diseases has unfortunately become a common disease and nutritional research in this field is my interest. The authors have written well to present their findings. | Thank you for this comment and for reviewing our manuscript. |  |
| - Please provide a table of how many food groups were included in the PCA and RRR? And what were the components of each group? | We have added this in a supplementary table. | S1table |
| - Please provide a table of factor loading of food groups in each pattern, like the reference below: https://link.springer.com/article/10.1186/s12889-022-13166-0 | We have added this in a supplementary table. | S2 table |

Thank you,

Kind regards,

Evangelista Malindisa (On behalf of all co-authors)
